# Risk Factors and Outcome of Sepsis in Traumatic Patients and Pathogen Detection Using Metagenomic Next-Generation Sequencing

**DOI:** 10.1155/2022/2549413

**Published:** 2022-04-25

**Authors:** Yiqing Tong, Jianming Zhang, Yimu Fu, Xingxing He, Qiming Feng

**Affiliations:** Department of Emergency Medicine, Shanghai Jiao Tong University, Affiliated Sixth People' Hospital, Shanghai, China

## Abstract

**Objective:**

Sepsis, a life-threatening clinical syndrome, is a leading cause of mortality after experiencing multiple traumas. Once diagnosed with sepsis, patients should be given an appropriate empiric antimicrobial treatment followed by the specific antibiotic therapy based on blood culture due to its rapid progression to tissue damage and organ failure. In this study, we aimed to analyze the risk factors and outcome of sepsis in traumatic patients and to investigate the performance of metagenomic next-generation sequencing (mNGS) compared with standard microbiological diagnostics in post-traumatic sepsis.

**Methods:**

The study included 528 patients with multiple traumas among which there were 142 cases with post-traumatic sepsis. Patients' demographic and clinical data were recorded. The outcome measures included mortality during the emergency intensive care unit (EICU), EICU length of stay (LOS), all-cause 28-day mortality, and total ventilator days in 28 days after admission. A total of 89 blood samples from 89 septic patients underwent standard microbiological blood cultures and 89 samples of peripheral blood (*n* = 21), wound secretion (*n* = 41), bronchoalveolar lavage fluid (BALF) (19), ascites (*n* = 5), and sputum (*n* = 3) underwent mNGS. Pathogen detection was compared between standard microbiological blood cultures and mNGS.

**Results:**

The sepsis group and non-sepsis group exhibited significant differences regarding shock on admission, blood transfusion, mechanical ventilation, body temperature, heart rate, WBC count, neutrophil count, hematocrit, urea nitrogen, creatinine, CRP, D-D dimer, PCT, scores of APACHE II, sequential organ failure assessment (SOFA), and Injury Severity Score (ISS) on admission to the EICU, and Multiple Organ Dysfunction Syndromes (MODS) (*P* < 0.05). Multivariate logistic regression analysis showed that scores of APACHE II, SOFA, and ISS on admission, and MODS were independent risk factors for the occurrence of sepsis in patients with multiple traumas. The 28-day mortality was higher in the sepsis group than in the non-sepsis group (45.07% vs. 19.17%, *P* < 0.001). The mortality during the EICU was higher in the sepsis group than in the non-sepsis group (*P*=0.002). The LOS in the EICU in the sepsis group was increased compared with the non-sepsis group (*P*=0.004). The total ventilator days in 28 days after admission in the sepsis group was increased compared with the non-sepsis group (*P* < 0.001). Multivariate logistic regression analysis showed that septic shock, APACHE II score on admission, SOFA score, and MODS were independent risk factors of death for patients with post-traumatic sepsis. The positive detection rate of mNGS was 91.01% (81/89), which was significantly higher than that of standard microbiological blood cultures (39.33% (35/89)). Standard microbiological blood cultures and mNGS methods demonstrated double positive results in 33 (37.08%) specimens and double-negative results in 8 (8.99%) specimens, while 46 (51.69%) samples and 2 (2.25%) samples had positive results only with mNGS or culture alone, respectively.

**Conclusion:**

Our study identifies risk factors for the incidence and death of sepsis in traumatic patients and shows that mNGS may serve as a better diagnostic tool for the identification of pathogens in post-traumatic sepsis than standard microbiological blood cultures.

## 1. Introduction

Trauma is a main cause of morbidity and mortality in most population worldwide. It has been reported various traumas led to 2.8 million hospitalizations and approximately 230,000 deaths annually, increasing huge healthcare costs in medical system all over the world [1]. Extensive injury to tissues and ischemia-induced release of damage-associated molecular patterns following severe trauma result in a robust inflammatory response, which is supposed to disrupt homeostasis of the immune system and affect the innate and adaptive arms of the immune system, leading to nosocomial infection, sepsis and Multiple Organ Dysfunction Syndromes (MODS) in the later stage [2, 3]. Sepsis is a complication caused by trauma and accounts for 10% of post-traumatic deaths [4]. Sepsis, as an extreme reaction of human response to infection, can quickly lead to life-threatening symptoms such as human tissue injury and organ failure if without timely treatment [5]. Bacterial infections are the most common cause of sepsis, but viruses and fungi may occur in patients with comorbid conditions and immunosuppression. The lower respiratory tract is the most common infection sites in hospitalized patients, followed by intra-abdominal, bloodstream, and urinary tract infections [6]. Major bloodstream isolates include *S. aureus*, *E. coli*, *Klebsiella spp.*, *Pseudomonas aeruginosa*, *Enterococci*, *Streptococci* and coagulase-negative *staphylococci* [7]. The Extended Prevalence of Infection in Intensive Care (EPIC III) study including 15,000 ICU patients from 88 countries reported 65% of patients had at least 1 positive microbiological culture with Gram-negative pathogens being most common, including *Klebsiella species, E. coli, Pseudomonas species, Enterobacteriaceae, Proteus, Stenotrophomonas, Serratia,* and *Acinetobacter* species [8]. Current therapies to treat sepsis mainly rely on supportive cares, such as antibiotics, intravenous fluids, and vasopressors [9]. Although organs damaged by Gram-positive sepsis exhibit no clinical difference from Gram-negative sepsis, Gram-positive bacterial sepsis relies on the production of exotoxin but the initiating factor of Gram-negative bacterial sepsis is endotoxin [10, 11]. Gram-positive pathogens need a highly orchestrated host response allowing intracellular killing by neutrophils and macrophages, whereas Gram-negative bacteria can be readily killed in the extracellular space by antibody and complement [12]. It is becoming increasingly important to understand the difference between Gram-positive and Gram-negative sepsis to deduce in which they initiate the disease and then to discover novel therapeutics due to the rising incidence of antibiotic resistant microbes.

Standard microbiological blood cultures can have variable yields, long turnaround times, and low sensitivity, which contribute to inappropriate antibiotic therapy [13]. Metagenomic next-generation sequencing (mNGS) provides a sensitive and thorough approach that allows detection of pathogens in clinical samples regardless of whether they are viral, bacterial, fungal, or parasitic [14]. The detection approach of mNGS has become increasingly available to identify pathogens in cases of various diseases such as central nervous system infection [15], tuberculous meningitis [16], and severe pneumonia [17], showing better sensitivity and specificity than conventional methods. Recent studies have indicated the application of mNGS as an adjunctive diagnostic tool for the identification of pathogens in patients with clinically sepsis. For example, Grumaz et al. revealed that it only took roughly 30 h to accomplish from sample preparation to species identification report, thus making this approach a promising diagnostic platform for critically ill patients with bloodstream infections than blood culture [18]. In addition to rapid detection, mNGS is also helpful to confirm the pathogens of severe sepsis when blood culture results were negative [19]. However, the application of mNGS for diagnosis of sepsis patients following trauma remains insufficient. In this study, we performed a comprehensive analysis of risk factors associated with morbidity and mortality in patients with post-traumatic sepsis and compared the diagnostic sensitivity between the mNGS and blood culture.

## 2. Methods

### 2.1. Study Population

A total of 528 patients with multiple traumas admitted to emergency intensive care unit (EICU) of Shanghai Sixth People's Hospital from January 2019 to September 2021 were included in this study. The inclusion criteria for patient recruitment were as follows: multiple traumas as a single cause for admission into the EICU; aged more than 18 years; admission within 24 h following trauma; the average Injury Severity Score (ISS) > 12 (two independent investigators used the ISS to rate severity of trauma). Those without complete clinical records and data, with injury caused by a knife or sharp or pointed instrument, burn injuries, chemically induced injury, during pregnancy and lactation, dead within 24 h after admission, with immunosuppression due to solid organ transplantation, HIV infection and chemoradiotherapy in recent 6 weeks, and suffering from craniocerebral trauma (Glasgow Coma Scale <8 scores) were excluded from the study. These 528 trauma patients were assigned into sepsis group (*n* = 142) and non-sepsis group (*n* = 386) according to the occurrence of sepsis. The diagnosis of sepsis was confirmed based on sequential organ failure assessment (SOFA) score from the 2016 international consensus for sepsis and septic shock [20], specifically with SOFA ≥ 2 for at least one of respiratory function (ratio of partial pressure of arterial oxygen and fraction of inspired oxygen (PaO_2_/FiO_2_)  < 300), liver function (bilirubin > 33 *μ*mol/L), coagulation (platelets < 100 × 10^3^/*μ*L), and renal function (creatinine > 171 *μ*mol/L) [21]. The study protocol was approved by the Ethics Committee of Shanghai Sixth People's Hospital and a signed informed consent form was received from each patient. If the patient failed to give consent because of the underlying severe infection, informed consent could be given by the patient's legal guardian until the patients were informed. All data were anonymized before analysis.

### 2.2. Data Collection

The demographic and clinical data of all patients were collected, including age, gender, BMI, causes and location of injuries, previous medical history of hypertension, coronary heart disease, diabetes mellitus, asthma, bronchiectasis, chronic obstructive pulmonary disease, chronic kidney disease, stroke, cirrhosis, gastrointestinal ulcer, undergoing craniocerebral, cardiac, chest, and abdominal surgery within 3 months, time of admission, shock on admission, receiving operation within 24 h after EICU admission, blood transfusion, mechanical ventilation, body temperature, heart rate, blood pressure, blood pH, oxygenation index, oxygen saturation of blood (SpO_2_), oxygen partial pressure (PaO_2_), carbon dioxide partial pressure (PaCO_2_), white blood cell (WBC) count, neutrophil count, hematocrit, platelet count, total bile acid (TBA), albumin, total bilirubin (TBIL), urea nitrogen, creatinine, C-reactive protein (CRP), D-D dimer, procalcitonin (PCT), lactic acid, blood glucose, ISS score, acute physiology and chronic health evaluation II (APACHE II) score, SOFA score on admission, MODS, pathogens, and dysfunction in more than one organ system.

The EICU outcome measures included mortality during the EICU and length of stay (LOS) in the EICU. Other outcome measures included all-cause 28-day mortality and total ventilator days in 28 days after admission.

The ISS scale is proposed by Baker et al. [22] according to the grading system of the Abbreviated Injury Scale (AIS) and classifies injury sites into six parts including head and neck, face, chest, abdomen and pelvis, limbs and pelvis, and body surface. The injury degree for each part is rated as six levels including mild, moderate, severe but not life-threatening, severe but life-threatening but survivable, extremely severe, and unable to rescue success, with scores ranging from 1 to 6 points. The effective range of ISS score was 1–75 points. Higher the score was, the more serious the injury and lower survival rate would be.

The APACHE II scale has been commonly applied as an index of illness severity in patients admitted to ICU and validated for many research and clinical audit purposes. The total scores comprised acute physiology score, age score, and chronic health status score. The APACHE II score was calculated according to clinical data and laboratory test indexes within 24 h following admission [23]. A higher score reflects a more critical condition, with a theoretical maximum score of 71.

The SOFA scores are calculated on admission to ICU and at every 24 h period to reflect the function of an organ system (respiratory, nervous, cardiovascular, liver, coagulation, and kidney) and allocate a score ranging from 0 to 4 [24]. The distribution of scores on SOFA is detailed in Table 1. A higher score is indicative of a more serious condition.

### 2.3. Standard Microbiological Blood Cultures

The Surviving Sepsis Campaign Bundle [25] reports indicators of blood culture: fever (body temperature ≥39.5°C) or body temperature ≥38.5°C, with a sepsis-related sign, such as shiver; more than 5 days of indwelling central venous catheters; white blood cell (WBC) count >1.8 × 10^9^; systolic blood pressure (SBP) < 90 mmHg; and unexplained infection. We collected three blood culture sets (the recommended volume of blood of 5–10 mL), each one consisting of a BACTEC Plus Aerobic/F bottle and a Plus Anaerobic/F medium bottle (Becton Dickinson Company, New Jersey, USA), the top of which were decontaminated with alcohol, from 89 septic patients who triggered the sepsis alert system (admitted into the EICU of our hospital from January 2020 to September 2021) in the sepsis group. The bottles were immediately transported to our laboratory for incubation and processed in an automated continuous monitoring blood culturing instrument (BACTEC FX, Becton Dickinson Company). Aliquots from each positive blood culture were Gram-stained and then subcultured on a chocolate blood medium (Becton Dickinson Company). The culture plate was read for their colony characteristic and the further biochemical test was run for the identification of specific microorganisms, as well as antibiotic susceptibility test was performed by disk diffusion technique. Only rapid examination by Gram-staining smear and standard microbiological blood culture results were available as microbiological evidence for choosing therapeutic interventions for sepsis.

### 2.4. Pathogen Detection by mNGS

We collected wound secretion, bronchoalveolar lavage fluid (BALF), ascites, and sputum from 89 septic patients and transported these samples into CapitalBio Corporation (Beijing, China) for mNGS detection and processing. In brief, DNA was extracted using the TIANamp Micro DNA Kit (DP316, Tiangen Biotech, Beijing, China), dissolved in tris-ethylenediaminetetraacetic acid buffer, and then evaluated for quantity and quality. The DNA libraries were constructed through DNA-fragmentation, end-repair, adapter-ligation, and polymerase chain reaction (PCR) amplification. Qualified libraries were subsequently sequenced on the BGISEQ-50 platform, and at least 20 M reads were obtained for each sample. High-quality sequencing data were generated, followed by computational subtraction of human host sequences that were mapped to the human reference genome (hg19). The remaining data were classified by simultaneous alignment to four microbial genome databases consisting of viruses, bacteria, fungi, and parasites. The classification reference databases were downloaded from NCBI (ftp://ftp.ncbi.nlm.nih.gov/genomes/). The following were the criteria for positive results of mNGS: (i) a species detected by mNGS with reads per million (RPM) ≥ 1 indicates a positive result for *Mycobacterium* and *Legionella pneumophila*; (ii) a species detected by mNGS with RPM ≥3 indicates a positive result for bacteria (excluding *Mycobacterium* and *Legionella pneumophila*) and virus with significantly different from the human genome sequence (such as adenovirus, influenza virus); and (iii) a species detected by mNGS indicates a positive result for RPM of fungi ≥5, RPM of parasites ≥10. The detection rate was compared between laboratory culture and mNGS. If the mNGS report and the blood culture report showed the same microorganism, the microorganism was confirmed. If the two reports revealed different results, anti-infection therapies were adjusted according to the mNGS report. The effective adjustment was confirmed if the patient clinical symptoms were improved.

### 2.5. Statistical Analysis

Given that the mortality of patients with multiple traumas (ISS >12) was 14.7% and the mortality of patients with multiple traumas followed by sepsis was 24.6%, we used PASS software to perform sample size power analysis, with *a* = 0.05, *β* = 0.10, and predicted loss rate of follow-up = 20%. After analysis, this study should recruit 216 cases, with 108 cases each for sepsis group and non-sepsis group. Given that the positive rate of blood culture in our hospital was 37% and the positive rate of mNGS was 68%, this study should include at least 44 cases for mNGS analysis, with *a* = 0.05 and *β* = 0.2 by using PASS software. The measurement data normally distributed were shown as mean ± standard deviation and compared using *t* test between two groups. The measurement data failing to normally distribute were shown as the median (interquartile range from 25% to 75%). The enumeration data were presented as percentage and compared using chi-square test. The possible influencing factors were included into the multivariate logistic regression model to analyze independent risk factors of sepsis in patients with post-traumatic infection. The Hosmer–Lemeshow test of goodness-of-fit was performed to examine model calibration. Data analysis was performed using SPSS 19.0 software package (IBM, USA). A *P*-value <0.05 reflects significant difference.

## 3. Results

### 3.1. Patient Characteristics between Sepsis and Non-Sepsis Groups

According to the occurrence of sepsis, 528 trauma patients were assigned into sepsis group (*n* = 142) and non-sepsis group (*n* = 386). Univariate analysis (Table 2) showed significant differences between the sepsis group and non-sepsis group with regard to shock on admission, blood transfusion, mechanical ventilation, body temperature, heart rate, WBC count, neutrophil count, hematocrit, urea nitrogen, creatinine, CRP, D-D dimer, and PCT (*P* < 0.05). Of note, the scores of APACHE II, SOFA, and ISS on admission to the EICU, and the proportion of patients with MODS in the sepsis group were higher than those in the non-sepsis group (*P* < 0.05). There was no significant difference in age, gender distribution, BMI, cause and location of trauma, previous medical history, time of admission, previous medical history, SBP, DBP, blood pH, oxygenation index, SpO2, PaO2, PaCO2, platelet count, TBA, albumin, TBIL, lactic acid, and blood glucose (*P* < 0.05).

### 3.2. Risk Factors for the Occurrence of Post-Traumatic Sepsis

Multivariate logistic regression analysis was performed by including shock on admission, blood transfusion, mechanical ventilation, body temperature, heart rate, WBC count, neutrophil count, hematocrit, urea nitrogen, creatinine, CRP, D-D dimer, PCT, APACHE II, SOFA and ISS on admission to the EICU, and MODS. It was showed that the APACHE II score on admission, SOFA score on admission, ISS score on admission, and MODS were independent risk factors for the occurrence of sepsis in multiple trauma patients (*P* < 0.05, Table 3). The predictive model satisfied the Hosmer–Lemeshow test for goodness-of-fit (*P*=0.63) and was therefore well-calibrated. We then analyzed the diagnostic performance of these risk factors for the occurrence of sepsis in multiple trauma patients. The diagnostic APACHE II score was 44.84 with a sensitivity of 78.17% and a specificity of 60.36%. The diagnostic SOFA score was 6.16 with a sensitivity of 80.28% and a specificity of 61.14%. The diagnostic ISS score was 21.45 with a sensitivity of 72.54% and a specificity of 57.77%. MODS showed a sensitivity of 71.83% and a specificity of 61.66%.

### 3.3. Risk Factors of Death for Patients with Post-Traumatic Sepsis

There were 64 cases of death (45.07%) in the sepsis group and 74 cases of death (19.17%) in the non-sepsis group in 28 days after admission. The 28-day mortality was higher in the sepsis group than in the non-sepsis group (*P* < 0.001). Death causes included primary disease, severe trauma, shock, multiple organ failure, blood infection, and AAD. More specifically, there were 38 cases of death (26.76%) during the EICU in the sepsis group and 56 cases of death (14.51%) during the EICU in the non-sepsis groups. The mortality during the EICU was higher in the sepsis group than in the non-sepsis group (*P*=0.002). The LOS in the EICU in the sepsis group was increased compared with the non-sepsis group (*P*=0.004). The total ventilator days in 28 days after admission in the sepsis group were increased compared with the non-sepsis group (*P* < 0.001, Table 4). According to the 28-day mortality, 142 patients with sepsis were sub-arranged into survivor group and non-survivor group. As shown in Table 5, significant differences were noted in the proportions of septic shock and mechanical ventilation, APACHE II score, SOFA score, ISS score, and MODS between survivor group and non-survivor group (*P* < 0.05). The 28-day mortality of patients with post-traumatic sepsis were regarded as dependent variables, septic shock, mechanical ventilation, APACHE II score on admission, SOFA score on admission, ISS score on admission, and MODS included in the logistic regression model as independent variables. Multivariate logistic regression analysis (Table 6) showed that septic shock, APACHE II score on admission, SOFA score, and MODS were independent risk factors of death for patients with post-traumatic sepsis. The predictive model satisfied the Hosmer–Lemeshow test for goodness-of-fit (*P*=0.69) and was therefore well-calibrated.

### 3.4. Pathogen Detection between Standard Microbiological Blood Cultures and mNGS

**Figure 1:**
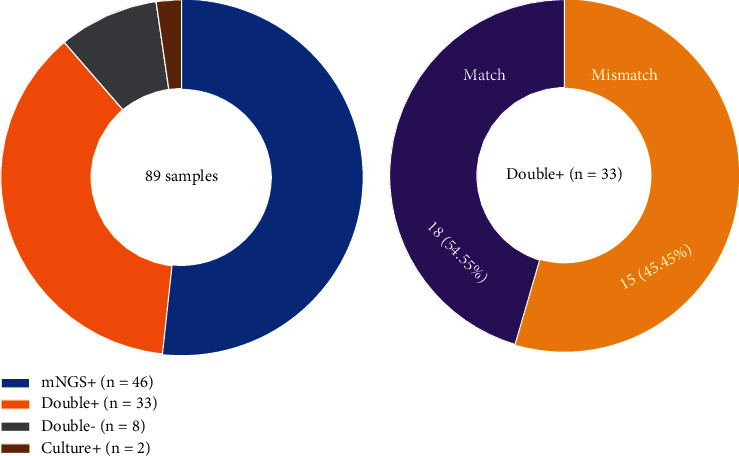
Comparison of pathogen detection between standard microbiological blood cultures and mNGS.

A total of 89 blood samples underwent standard microbiological blood cultures and 89 samples of peripheral blood (*n* = 21), wound secretion (*n* = 41), bronchoalveolar lavage fluid (BALF) (19), ascites (*n* = 5), and sputum (*n* = 3) underwent mNGS. The positive detection rate of mNGS was 91.01% (81/89), which was significantly higher than that of blood culture in our laboratory, 39.33% (35/89) (*P* < 0.001 by chi-square test). As shown in Table 7, the several common pathogens by mNGS and standard microbiological blood cultures were *Klebsiella pneumonia* (18 (20.22%) vs. 9 (10.11%)) followed by *Acinetobacter baumannii* (15 (16.85%) vs. 8 (8.99%)), *Staphylococcus* (11 (12.36%) vs. 5 (5.62%)), and *Pseudomonas aeruginosa* (8 (8.99%) vs. 4 (4.49%)). Of note, the mNGS method demonstrated an evidently higher positive rate than standard microbiological blood cultures regarding *Corynebacterium* detection. A total of 9 (10.11%) samples were noted to have viral infection, which was only identified by mNGS. Standard microbiological blood cultures and mNGS methods demonstrated double positive results in 33 (37.08%) specimens and double-negative results in 8 (8.99%) specimens, while 46 (51.69%) samples and 2 (2.25%) samples had positive results only with mNGS or culture alone, respectively. Among the specimens that had positive results from both methods, 18 (54.55%) were completely matched, while a mismatch was observed in 15 (45.45%) cases (Figure 1). The read values of *Klebsiella pneumonia*, *Acinetobacter baumannii*, *Staphylococcus*, *Pseudomonas aeruginosa*, and *Corynebacterium* were 12,272.5 (4,512, 75,362), 10491 (4,143, 42,626), 27,267 (6,372, 119,003), and 10293 (5,100, 43,246), respectively.

## 4. Discussion

Multiple traumas often lead to sepsis, which is a major reason causing death in non-cardiac ICU, accounting for 30.9% in-hospital mortality [26]. In this study, we not only analyzed risk factors for the occurrence of post-traumatic sepsis and mortality of patients with post-traumatic sepsis but also performed a comprehensive analysis on the mNGS for the etiological diagnosis of septic patients.

The present study initially found APACHE II score on admission, SOFA score on admission, ISS score on admission, and MODS were independent risk factors for the occurrence of sepsis in multiple trauma patients. Septic shock, APACHE II score on admission, SOFA score, and MODS were independent risk factors of death for patients with post-traumatic sepsis. ISS is the most commonly used tool for stratification of injured patients and have been widely used in trauma evaluation, which presents excellent performance in predicting mortality caused by blunt trauma when the patients' ISS are lower than 25 [27]. In 1999, SOFA was applied to evaluate organ dysfunction in trauma patients and associated with prediction of prolonged ICU stay or death [28]. The Sepsis-3 identified SOFA as the new scoring system to quantify organ dysfunction in sepsis patients and demonstrated that SOFA has advantages over other scoring systems in predicting overall prognosis in relation to mortality [20, 29]. This demonstration was similar to ours suggesting that SOFA score is an independent risk factor of death for patients with post-traumatic sepsis. The results in our study also indicated that septic shock, APACHE II, and MODS were closely related to mortality in sepsis patients following trauma. MODS is a symptom of two or more organ failure, which usually occurs after life-threatening physiological injury. Shock, sepsis, and insufficient tissue perfusion are the most common risk factors leading to MODS [30]. The presence of MODS increased the risk of death in patients with sepsis [31]. The APACHE II scoring system accurately measures the severity of patients and is closely related to the prognosis of critically ill patients [32, 33]. A previous report by Xie et al. showed septic patients had a high ICU mortality rate, sharing the same clinical characteristics with sepsis patients in our study [34].

The conventional culture process requires up to 48–72 h for a detailed analysis, meaning the selection of appropriate empiric antibiotic therapy can be delayed. Our findings suggested that the mNGS can identify multiple pathogens in clinical specimens including blood, wound secretion, BALF, ascites, and sputum from septic patients and shows evidently higher positive rates than diagnostics based on standard microbiological blood cultures in terms of timely and accurately determining etiological pathogens for suspected and confirmed cases of sepsis due to well-performed data interpretation. These benefits were also reported in previously reported studies [35, 36], which suggested that mNGS confers a valuable diagnostic platform for determining relevant pathogens mNGS is broadly applied for detecting pathogens and especially for the timely and accurate diagnosis of critical illness including sepsis due to suspected etiology microbes [37, 38]. Previous evidence has proved the application of mNGS in identifying various viruses via samples of nasopharyngeal swabs, serum, or solid tissue [39, 40], as well as in identification of bacteria from urine, vaginal swabs, or sputum [41, 42]. The present study successfully demonstrated the advantage of mNGS in diagnosis of pathogens through blood, wound secretion, BALF, ascites, and sputum samples from patients with post-traumatic sepsis. It was found that the positive detection rate of pathogens (91.01%) using mNGS was significantly higher than that (39.33%) through conventional blood cultures in our laboratory. A clinical study of the patients with severe pneumonia indicated that 85% patients were identified as pathogen positive in BALF samples by mNGS, and by contrast, conventional microbial tests only confirmed 50% patients as pathogen-positive [17]. Another study reported a case of severe sepsis patient and found the pathogen was negative following antibiotic treatment. However, *Streptococcus suis* infection was confirmed as a pathogen by mNGS and Sanger sequencing [19]. We further compared the diagnostic performance between mNGS and conventional culture procedures for identifying different pathogens. The mNGS showed higher positive rates in common pathogens for the development of sepsis compared with conventional culture procedures. As shown by our results, the several common pathogens by mNGS and standard microbiological blood cultures were *Klebsiella pneumonia* (18 (20.22%) vs. 9 (10.11%)) followed by *Acinetobacter baumannii* (15 (16.85%) vs. 8 (8.99%)), *Staphylococcus* (11 (12.36%) vs. 5 (5.62%)), and *Pseudomonas aeruginosa* (8 (8.99%) vs. 4 (4.49%)). A lower detection rate of these commonly identified pathogens as etiological microorganisms for septic patients in ICUs by could-based diagnostics revealed that Gram-negative organisms were the main cause for the development of in-hospital sepsis [34, 43]. The mNGS has been shown to identify many pathogens with negative results by standard microbiological blood cultures, as reflected by our results that *Corynebacterium, Candida albicans, Clostridium perfringens, Vibrio vulnificus,* and *Bacillus cereus* were identified by mNGS, while negative results of these were confirmed by standard microbiological blood cultures. Concurring with the study performed by Duan et al. [44], they demonstrated that mNGS had a higher sensitivity than the conventional cultures, especially in blood, BALF, and sputum samples. Positive results (negative results by the conventional cultures) and more common pathogen detection were associated with hospital stay and 28-day-mortality of adult patients with infections. Additionally, the read values of *Klebsiella pneumonia*, *Acinetobacter baumannii*, *Staphylococcus*, *Pseudomonas aeruginosa*, and *Corynebacterium* were 12,272.5 (4,512, 75,362), 10491 (4,143, 42,626), 27,267 (6,372, 119,003), and 10293 (5,100, 43,246), respectively. Currently, the read values of mNGS are commonly used for identification of distinct pathogens after optimization [45, 46]. However, cut-off reads for diagnosing distinct pathogens by mNGS and their clinical applications in septic patients remain unclarified.

Of note, several limitations should be taken into consideration when interpreting our results. First, this study was conducted by means of retrospective analysis, which limited comprehensive data analysis and further information on the use of antibiotics. Second is the absence of a relationship between the read values and prognoses of septic patients in this observation due to the relatively small sample size of patients with distinct pathogen infections. Further investigations with more clinical samples for mNGS detection will be performed in a prospective study for assessing mNGS in pathogen detection and antibiotic administration in septic patients from the ICU.

In summary, this single-center study, on the one hand, demonstrated that APACHE II score on admission, SOFA score on admission, ISS score on admission, and MODS may be associated with the occurrence of sepsis in multiple trauma patients. On the other hand, the findings in this study support that mNGS can identify multiple pathogens including common and rare pathogens in clinical specimens from septic patients, suggesting timely and accurate determination of etiological pathogens conferred by mNGS than diagnostics based on standard microbiological blood cultures.

## Figures and Tables

**Table 1:** Sequential organ failure assessment (SOFA).

Organ system	0	1	2	3	4
Respiratory: PaO2/FIO2 (mmHg)	> 400	≤400	≤300	≤200^*∗*^	≤100^*∗*^
Renal: Creatinine (mg/dl) or urine output	<1.2	1.2–1.9	2.0–3.4	3.5–4.9 or <500 ml/d	≥5.0 or <200 ml/d
Hepatic: Bilirubin (mg/dl)	<1.2	1.2–1.9	2.0–5.9	6.0–11.9	≥12.0
Cardiovascular: Hypotension	No hypotension	MAP<70 mmHg	Dopamine≤5 or dobutamine (any dose)^#^	Dopamine>5 or epinephrine≤0.1 or norepinephrine≤0.1#	Dopamine>15 or epinephrine>0.1 or norepinephrine>0.1^#^
Hematologic: Platelet count (×103/mm3)	> 150	≤150	≤100	≤50	≤20
Neurologic: Glasgow coma score	15	13–14	10–12	6–9	<6

^*∗*^With ventilatory support; ^#^Adrenergic agents administered for at least 1 h (doses given are in µg/kg/min).

**Table 2:** Patient characteristics between sepsis and non-sepsis groups.

Characteristic	Sepsis (*n* = 142)	Non-sepsis (*n* = 386)	*P*
Age (year)	50.18 ± 13.68	48.93 ± 14.34	0.369
Gender (male/%)	105 (73.94%)	281 (72.80%)	0.792
BMI	25.22 ± 3.60	24.70 ± 2.84	0.084
Cause of trauma (n/%)			0.093
Traffic-related injury	65 (45.77%)	190 (49.22%)	
Falling injury	54 (38.03)	163 (42.23%)	
Blunt-force injury	14 (9.86%)	20 (5.18%)	
Other	9 (6.34%)	13 (3.37%)	
Location of trauma			>0.05
Head and neck	74 (45.68%)	190 (49.22%)	
Limbs and pelvis	79 (55.63%)	178 (46.11%)	
Chest	97 (68.31%)	236 (61.14%)	
Abdomen	26 (18.31%)	59 (15.28%)	
Previous medical history (n/%)	22 (15.49%)	40 (10.36%)	0.104
Time of admission (h)	10.39 ± 5.33	10.08 ± 5.26	0.550
Shock on admission (n/%)	63	104	<0.001
Operation within 24 h after EICU admission (n/%)	110 (77.46%)	281 (72.80%)	0.278
Blood transfusion (n/%)	126 (88.73%)	281 (72.80%)	<0.001
Mechanical ventilation (n/%)	129 (90.85%)	300 (77.72%)	<0.001
Body temperature (°C)	37.59 ± 0.62	37.37 ± 0.42	<0.001
Heart rate (/min)	103.81 ± 17.09	92.00 ± 17.03	<0.001
SBP (mmHg)	122.47 ± 13.42	121.44 ± 13.48	0.143
DBP (mmHg)	68.63 ± 8.92	70.07 ± 8.22	0.082
Blood pH	7.39 ± 0.11	7.40 ± 0.09	0.288
Oxygenation index (mmHg)	314.14 ± 190.34	335.70 ± 198.59	0.264
SpO_2_ (%)	87.57 ± 12.6	88.22 ± 11.71	0.580
PaO_2_ (mmHg)	140.93 ± 65.82	139.1 ± 66.14	0.778
PaCO_2_ (mmHg)	37.11 ± 9.24	35.86 ± 6.22	0.076
WBC count (1 × 10^9^/L)	13.45 ± 4.84	11.43 ± 5.73	<0.001
Neutrophil count (1 × 10^9^/L)	14.21 ± 7.07	12.43 ± 6.5	0.007
Hematocrit (%)	31.49 ± 12.12	35.77 ± 14.95	0.002
Platelet count (1 × 10^9^/L)	165.59 ± 87.09	157.89 ± 71.22	0.301
TBA (*μ*mol/L)	18.14 ± 1.89	17.99 ± 2.24	0.478
Albumin (g/L)	33.26 ± 8.14	32.09 ± 7.28	0.114
TBIL (mg/dL)	20.48 ± 2.17	20.29 ± 1.94	0.335
Urea nitrogen (mmol/L)	7.35 ± 4.3	5.85 ± 2.06	<0.001
Creatinine (*μ*mol/L)	91.09 ± 47.85	73.41 ± 22.62	<0.001
CRP (mg/dL)	12.1 ± 0.78	7.10 ± 0.66	<0.001
D-D dimer (mg/L)	3.26 ± 1.27	1.22 ± 0.42	<0.001
PCT (mg/L)	4.03 ± 1.29	1.32 ± 0.18	<0.001
Lactic acid (mmol/L)	5.32 ± 1.72	5.09 ± 1.6	0.152
Blood glucose (mmol/L)	8.85 ± 3.32	8.4 ± 2.62	0.105
APACHE II score	51.52 ± 7.11	43.71 ± 6.32	<0.001
SOFA score	7.77 ± 2.13	5.36 ± 2.37	<0.001
ISS score	24.73 ± 4.92	20.43 ± 3.97	<0.001
MODS	102 (71.83%)	148 (38.24%)	<0.001
Respiratory dysfunction	115 (80.99%)	203 (52.59%)	<0.001
Coagulation disorder	66 (46.48%)	146 (37.82%)	0.072
Neurological dysfunction	55 (38.73%)	124 (32.12%)	0.155
Circulating dysfunction	51 (35.92%)	39 (10.10%)	<0.001
Urinary dysfunction	34 (23.94%)	19 (4.92%)	<0.001

SpO_2_, oxygen saturation of blood; PtO_2_, oxygen partial pressure; PtCO_2_, carbon dioxide partial pressure; Hb, hemoglobin; TBA, total bile acid; TBIL, total bilirubin; CRP, C-reactive protein; PCT, procalcitonin; APACHE II, acute physiology and chronic health evaluation II; SOFA, sequential organ failure assessment; ISS, Injury Severity Score; MODS, Multiple Organ Dysfunction Syndromes. The measurement data which are normally distributed are shown as mean ± standard deviation and compared by student's *t*-test. The enumeration data are presented as percentage and compared using chi-square test.

**Table 3:** Multivariate logistic regression analysis of independent risk factors for the occurrence of sepsis in multiple trauma patients.

Variables	Assignment	OR (95%CI)	*P*
APACHE II score	Actual value	1.260 (1.183–1.342)	<0.001
SOFA score	Actual value	1.745 (1.456–2.092)	<0.001
ISS score	Actual value	1.361 (1.234–1.502)	<0.001
MODS	(Yes = 1 or No = 0)	5.382 (2.501–11.582)	<0.001

APACHE II, acute physiology and chronic health evaluation II; SOFA, sequential organ failure assessment; ISS, Injury Severity Score; MODS, Multiple Organ Dysfunction Syndromes.

**Table 4:** Patient outcomes between sepsis and non-sepsis groups.

Outcome	Sepsis (*n* = 142)	Non-sepsis (*n* = 386)	*P*
Death during the EICU (n/%)	38 (26.76%)	56 (14.51%)	0.002
LOS in the EICU (d)	19.35 ± 21.16	13.80 ± 18.54	0.004
Total ventilator days (d)	9.51 ± 10.66	4.56 ± 5.92	<0.001
28-day mortality ((n/%))	64 (45.07%)	74 (19.17%)	<0.001

EICU, emergency intensive care unit; LOS, length of stay.

**Table 5:** Patient characteristics between survivor group and non-survivor group.

Characteristic	Assignment	Sepsis (*n* = 64)	Non-sepsis (*n* = 78)	*P*
Septic shock	(Yes = 1 or No = 0)	38 (59.38%)	25 (32.05%)	0.001
Mechanical ventilation (n/%)	(Yes = 1 or No = 0)	62 (96.88%)	67 (85.90%)	0.038
APACHE II score	Actual value	54.20 ± 6.74	49.32 ± 6.68	<0.001
SOFA score	Actual value	8.43 ± 2.17	7.23 ± 1.96	<0.001
ISS score	Actual value	26.12 ± 4.47	23.59 ± 5.01	0.002
MODS	(Yes = 1 or No = 0)	58 (90.63%)	44 (56.41%)	<0.001

APACHE II, acute physiology and chronic health evaluation II; SOFA, sequential organ failure assessment; ISS, Injury Severity Score; MODS, Multiple Organ Dysfunction Syndromes.

**Table 6:** Multivariate logistic regression analysis of independent risk factors for the death for patients with post-traumatic sepsis.

Variables	*P*	OR (95%CI)
Septic shock	0.025	2.749 (1.138–6.643)
APACHE II score	<0.001	1.127 (1.054–1.205)
SOFA score	0.002	1.424 (1.135–1.787)
MODS	0.005	4.683 (1.581–13.872)

APACHE II, acute physiology and chronic health evaluation II; SOFA, sequential organ failure assessment; MODS, Multiple Organ Dysfunction Syndromes.

**Table 7:** Microbial diversity detected by mNGS.

Pathogen	mNGS	Blood culture
*Klebsiella pneumonia*	18	9
*Acinetobacter baumannii*	15	8
*Staphylococcus*	11	5
*Pseudomonas aeruginosa*	8	4
*Corynebacterium*	7	0
*Escherichia coli*	6	3
*Enterococcus faecalis*	5	2
*Candida albicans*	5	0
*Streptococcus pneumonia*	4	2
*Clostridium perfringens*	4	0
*Vibrio vulnificus*	4	0
*Enterobacter cloacae*	3	1
*Bacillus cereus*	3	0
*Hemophilus influenzae*	1	1
Virus	9	0

## Data Availability

The data used to support the findings of this study are included within the article.
